# Values and vendettas: Populist science governance in Mexico

**DOI:** 10.1177/03063127221140020

**Published:** 2022-12-05

**Authors:** Luis Reyes-Galindo

**Affiliations:** Wageningen University and Research, Wageningen, The Netherlands

**Keywords:** populism, post-truth, Mexico, science governance

## Abstract

This article aims to diversify STS perspectives on populism by addressing a sequence of episodes of Mexican science policy in terms of clashes between populism and scientific communities. The article describes a reorientation of Mexican science policy that has destabilized the academic system during the present administration. Specifically, it looks at the legislative project initiated by Mexico’s National Science and Technology Council (Conacyt) to overhaul the national regulatory framework on science, technology and innovation, and controversial political actions taken by Conacyt against the scientific community. Contextualizing these grievances, the article concludes that at stake is a form of ‘trickle-down populism’ that, through systematic authoritarianism, seeks to impose on the academic community a model of ‘populist science governance’.

El político, conscientemente, no obra nunca contra su interés. ¿Qué puede entonces agradecerse?^1^

- M. L. Guzmán, *La Sombra del Caudillo*

## Introduction: Populism in Science and Technology Studies

Confrontations between science and populism have recently garnered considerable attention from Science and Technology Studies (STS) focusing on the consequences of a ‘post-truth’ political turn in the Global North (Fujimura & Holmes, 2019; Holman, 2020; Kelkar, 2019; Lynch, 2020; Sismondo, 2017 and essays therein). Yet, as Rovira Kaltwasser et al. (2017) point out, researchers have long recognized in ‘populism’ a diversity of styles of government that transcends STS’s mostly US-centric perspective. As scholars of populism also warn, ‘the concept of populism is indeed helpful to understand contemporary politics’, but a shallow use of the concept runs the risk of ‘a diverse number of academics and pundits … using it mainly as a buzzword’ (Mudde & Rovira Kaltwasser, 2018, pp. 1667–1693).

In this article I aim to diversify STS perspectives on populism by addressing recent developments in Mexican science policy, taken as a case study of clashes between populists in positions of power and non-populists in scientific communities. I describe a reorientation of Mexican science policy during the present administration, which has been perceived by many Mexican scientists as disruptive and damaging to the country’s academic/scientific system. I narrate the lead-up to the legislative project, initiated by Mexico’s National Science and Technology Council (Conacyt), which sought to overhaul the national regulatory framework on science, technology, and innovation (STI).^2^ I then describe parallel actions taken by Conacyt that were defended as advances of the federal government’s STI agenda: policy decisions that sought to centralize power and control of the STI system to install, despite significant opposition from vast sectors of the scientific community, serial institutional violence and the delegitimization of actors not aligning with Conacyt’s agenda – acting as proxy of the federal government. Thus, while noting that some of the actions promulgated by Conacyt documents might be read as solid anchors for desirable and legitimate institutional transformations of the Mexican STI system, the mismatch between discourse and acts of institutional violence is the central focus here – the type of ‘doublespeak’ that Lutz (1989) identifies as couched in euphemism used to make institutional violence palatable in and for the public eye.

To clarify, while there are voices in Mexican science that support Conacyt policies, there are marked differences between the overwhelmingly antagonistic members of the natural sciences and pro-Conacyt sections of the humanities and the social sciences. At the same time, prominent academic voices supporting Conacyt, particularly in the humanities, come in two distinct flavors. On the one hand, there is a documented rise of academics-turned-political figures within the ruling party who have become mouthpieces of government policy, but despite their academic origin can hardly be taken to represent wider academic sentiment (Sheridan, 2022). In contrast, there are also distinguished scholars who, while openly aligned with the political project of the current administration, have remarked that changes to the internal politics of Mexican academia are both necessary and a historical duty, but still denounce the graver cases of institutional violence.^3^ I use the term ‘scientific community’ mainly to refer to Mexican natural scientists, but with keen awareness that the term encompasses a diverse collectivity of both research- and education-focused academics who, despite this diversity, have been increasingly unified by their dissatisfaction with Conacyt policy.

## The banner of Mexican scientific populism: The anti-neoliberal State

To understand how Conacyt’s actions can be conceptualized as an offshoot of the federal government’s populist ‘style’ (Moffitt & Tormey, 2014), I make use of Levitsky and Loxton’s (2013, pp. 107–108) observation that a central feature of many contemporary Latin American governments generally classified as populist is their tendency to conjugate, firstly, weak democratic institutions; and secondly, ‘competitive authoritarianism’ – or, ‘electoral regimes in which widespread incumbent abuse skewed the playing field against opponents … to such an extent that the opposition’s ability to compete is seriously compromised’. The general tone of Latin American populist is thus a push for the centralization of power and the weakening of existing ‘corrupt’ institutions as a means to push an agenda, legitimate or not, ‘for the people’ and against the people’s – and, by proxy, the government’s – political adversaries. As such, many populist Latin American governments, while originally aligning with social justice movements, often end up acting out totalitarian attitudes that include ‘surveillance and blackmail; “legal” persecution for defamation, tax violations, or corruption; attacks by government-sponsored mobs; and occasional arrest or exile’ (Levitsky & Loxton, 2013, p. 108).

The core contribution of this article is to illustrate how such authoritarian tactics have been serially enacted by Conacyt authorities to affect critics of the government’s projected science policy, being furthermore accompanied by unilateral governance decisions that ignored community sentiment and have already negatively affected scientific communities. The populist attitude is found not only at the federal level but has now also become the primary style to implement science policy in and of itself. Thus, while the conjugation of political discourse that vindicates legitimate ‘popular’ demands, and also institutional violence against political enemies, has been a structuring feature of Latin American populisms, I argue that they can also, as in the case of Conacyt, ‘trickle down’ into a form of *populist science governance*.

The article focuses on a set of new guiding ‘principles’ that are claimed to anchor STI policy interventions, these being consistently repeated in preambles to Conacyt legal documents, briefs and official communications since 2019, when the recent administration took over. Institutional documents are thus used as resource material for an initial ‘natural system’ organizational analysis (Scott & Davis, 2007). This includes the ‘scrutiny of organizational cultures, values or identities’, as used in comparative international analyses of scientific and higher education institutions to sensitize analysis ‘to the disparity between goals as embedded in policy and goals embedded in practice’ (Wittrock et al., 2021, p. 11). Additionally, I consider critical outlooks, such as discussions on how values cannot be reduced to ‘analyzing political documents or doing value preference surveys or other forms of asking stakeholders directly about their “values”’, but rather require a practice-based approach in which ‘the active realization of values in practices center stage’ (Boenink & Kudina, 2020).

In addition to the documentary analysis, to better understand both ‘pro’ and ‘anti’ Conacyt positions, in the early stages of this research I carried out a full monitoring of two independent forums – held over two weeks in 2021 and totaling over 50 hours of live discussions – both of them focused on Conacyt’s legal framework initiatives, and on the general state of STI and higher education in the country. During the first week, in an event with an explicitly critical undertone organized by Mexico’s most important higher education institutions, natural scientists and others clearly expressed their most pressing concerns: the centralization of power and the unrepresentativeness of scientific voices in policy decisions, funding woes and the elimination of specific funding instruments, and changes to ‘traditional’ scientific drivers and values. The positions expressed were, unsurprisingly given the choice of participants and the organizers’ outlook, overwhelmingly negative towards Conacyt, though participants admitted that reforms to the scientific governance system could be beneficial if direct state interference were avoided.^4^ The second week-long discussion forums were organized by Conacyt itself, with a more prominent inclusion of humanities and social science academics and Conacyt political sympathizers.^5^ In this second forum, legal scholars defended the changes as improvements on the organizational aspects of the new STI Law, while various social sciences and humanities academics praised the inclusion of epistemic equality and related values into policy. Members of the natural sciences, while less prominent in this second forum, largely echoed the same sentiments as in first previous event. The forums thus confirmed a clear division between a Conacyt-critical natural sciences community and the more Conacyt-supporting, but divided, humanities and social sciences communities.

Other sources include a review of email discussions lists of Mexican academic networks, over two years of monitoring Twitter material related to Mexican STI and Conacyt, a review of national and international news coverage, analysis of online videos and documents compiled by Conacyt as support for the initial drafts of the law, a limited number of interviews with early career scholars on their outlook of the STI system, and finally, Conacyt official statements and press bulletins.

While preambles to Conacyt’s official statements frame science as a tool for the state to consolidate its electorally mandated project of increasing social justice through the new ‘principles’ – mainly, (epistemic) equity and non-discrimination, inclusion, plurality, solidarity, social good, and precaution – independent analyses argue that these have done little to inform the *contents* of policy. Instead of a pluralist law, critics perceive Conacyt as promoting a vertical and centralized governance framework that excludes outside input. Following the pattern that Levitsky and Loxton (2013) note, in parallel to the centralization of power, the impending crisis peaked when these controversial governance decisions were contested by sectors of the scientific community and, in response, Conacyt set up a series of severe political attacks on academics unaligned with ‘the people’s’ interests, over which the federal government claims unique representation. Thus, although the present administration’s left-leaning positioning is different from other populist sites in the Americas governed by hard right-wing populists, it also shares worrying similarities with the ‘war on science’ denounced by Brazilian STS scholars during the Jair Bolsonaro presidency, and to anti-scientific Trump-like ‘post-truth’ discourse (Duarte, 2020; Monteiro, 2020; Reyes-Galindo, 2021).

I argue that populism, as enacted by Conacyt, has visibly eroded and negatively affected Mexican academic life, particularly for more vulnerable scholars and early career researchers, despite the discursive shroud of social advancement. Framed as a fight against ‘neoliberal science’, following the federal governments’ agenda of organic change against the previous administrations, Conacyt director Álvarez-Buylla (2021) explained in an opinion piece:

We consider it is not only unacceptable, but indeed dangerous, to make of science a merchandise or a superfluous luxury good. It is now the time to turn it to public service, which can only acquire its maximal value as a tool for transformations oriented towards social wellbeing and environmental care. … Despite the many crises our country has suffered through, mainly through the adoption of the neoliberal credo by the political establishment that usurped popular representativity, there is a consolidated scientific community in Mexico with more than 70,000 members from across all social strata. … In the context of transformation that the Mexican people mandated with their vote on 1^st^ July 2018, Conacyt has begun a concentrated effort to be rid of the weights left behind by the neoliberal regime.

While this stance against ‘neoliberal’ science at first glance appears to resonate with well-known STS and social critiques of commercialized science (Fernández Pinto, 2017, 2021; Lave et al., 2010; Mirowski, 2011; Moore et al., 2011), we should be concerned popular discourse that claims to ‘to render scientific knowledge more accessible to the general public and research more responsive to the wishes of the scientist’ yet ‘turns out to be diversionary tactics and irrelevant conceits’ (Mirowski, 2018, p. 193).

Indeed, as described in numerous journalistic and scientific outlets (e.g. Gutiérrez Jaber, 2021; Pérez Ortega & Gutiérrez Jaber, 2021; Pérez Ortega & Wessel, 2020; Reardon, 2021; Vidal Valero, 2021), Mexico’s natural sciences community, despite some organized pushback to Conacyt interventions, has witnessed during the past three years a series of reforms to organizational bylaws, procedures and funding channels that have affected Mexican academia at large, crippling various established programs at all professional and higher-educational levels. Recently unionized Conacyt early career researchers, for example, have documented the impact of such policy choices, which have been immediately felt by junior academics through increasing precariousness and labor insecurity (see Siintracatedras, 2022). While the defunding of vast swathes of research areas and the elimination of postgraduate mobility programs has led senior scientists to speak of a ‘lost generation’ of researchers in the longer term, recent research on the participation of Latin America in international funding programs has already shown some of the effects of defunding decisions. In terms of European cooperation, for example, ‘Mexico is the clear loser’ among the five largest Latin American participants in the EU’s flagship Horizon2020 program, which was explained by a Conacyt ‘diplomat’ as due to Mexico ‘not having availability for funds that the new rule introduced in H2020 entailed’ (Uribe-Mallarino, 2022, p. 28).

## Setting the Mexican STI system in a Latin American context

Mexico is a country at the margins of the highly developed scientific world, its relationship to the Anglo-European scientific establishment mainly one of coloniality (Harding, 2011; Mignolo, 2007; Seth, 2009) and dependence (Dagnino et al., 1996). Characteristic of Latin America, Mexico has a relatively young scientific system compared with the powerhouses of the Global North, with professionalization of science occurring only as the 20th century progressed (Alonso-Pavón et al., 2020). As Kreimer (2019, p. 12) remarks, coloniality encompasses ‘a certain relative weakness of locally produced science’ paired with a ‘state decision-making machinery itself, which generally does without any locally produced knowledge’. Thus, in Latin American STS there is a concern with fostering institutional scientific and technological development that is historically based on ‘inquiries centered around how to make S&T contribute to the development of their societies’ (Kreimer & Vessuri, 2018, p. 21). This contrasts with foundational Anglo-European STS projections, which Kreimer and Vessuri (2018, p. 22) recognize as stemming from social movements that ‘challenged the idea of science’s autonomy by highlighting the internal barriers within the laboratory that produce individualistic and elitist scientific practices, or between the laboratory and the outside world’.

Another remarkable difference between Latin American and Anglo-European scientific systems is the lack of cohesion between state, private and public interests in science and technology (Arvanitis & Dutrénit, 1997; Tamayo Rodrígez & Bojorquez Bojorquez, 2021), so that, ‘science and technology have not finalized the consolidation of relevant political spaces in political classes’ priorities’ (Mercado & Casas, 2016, p. 25). Lacking sustained private-sector involvement (Rivas Tovar, 2004), the Mexican federal government has turned into the almost sole, even if insufficient, source of scientific funding, in stark difference to many parts of the Global North (Fernández Pinto, 2021). While this provides a funding lifeline, it also makes Mexican science highly vulnerable to the influence of immediate political interests, with sustained funding lines often limited to ‘cases in which science … created a realm of its own within the local culture by relating research activities to particular issues concerning the country’s interests’ (Cueto, 2021, p. 235). Government interventions in scientific funding are thus also deep political interventions within Latin American science governance.

Finally, one must also consider the larger instability characteristic of Latin American democracies. Given regional histories of totalitarianism (both militarized and non-military one-party systems were the norm until a few decades ago), autonomy and academic freedom are, unsurprisingly, political values strongly orienting Latin American science. On the one hand, both are understood as a means for protection from government abuse, interference and censorship (Schoijet & Worthington, 1993). On the other, as Herrera (2015) points out, autonomy is used by states to manage disinterest in science (Casas & Mercado, 2016; Vessuri, 2007). Recent multi-national studies of institutional values in research reaffirm that autonomy remains a fundamental yet conflicting value for Latin American science policy (Reyes-Galindo et al., 2019; Wittrock et al., 2021), making scholars feel safe in the sphere of autonomous ‘objective science’, while allowing states to minimize their support of scientific sectors devoid of immediate political rewards – or what Neves (2022) has referred to as ‘the regime of the management of irrelevance’ in peripheral science contexts.

## A short introduction to Mexican post-2018 politics

The backdrop to the present situation is the sweeping, definitive electoral win by the recently created *Morena* political party in the 2018 Mexican federal elections. Led by current president Andrés Manuel López Obrador (widely known as AMLO), Morena also established a majority alliance in both legislative chambers. Morena’s political power is thus only curbed, within the division of powers, by the judiciary, and a weakened opposition in the hands of the outgoing centrist PRI (*Partido Revolucionario Institucional*), the arch-nemesis right PAN (*Partido Acción Nacional*) party, and the tattered remains of the nearly defunct left PRD (*Partido de la Revolución Democrática*). Despite often painting itself as an entirely new political force, Morena’s core is built on the 2010s PRD’s disintegration into rival factions, with AMLO’s group transforming itself into a separate political party, Morena. In this transition, Mexican politics has seen in AMLO (a former high-ranking PRI member, PRD Mexico City mayor, and twice PRD Presidential candidate) the establishment of ‘strongman’ politics coupled with a compliant legislature. Thus, although separation of powers is an organizing hallmark of the Mexican political system and its primary form of checks and balances, in reality the balance of power is currently tightly gripped across two of the three powers under a single party banner led by a clear party leader.

A marked difference between Morena and the two PAN (2000–2012) and PRI (2012–2018) post-transition presidencies is its socially progressive discourse, backed by targeted social aid programs, a significant rise of the minimum wage, and reforms to major national laws. Conversely, other government actions such as the full militarization of the national police force, a preference for mega-infrastructure projects tied to developmental agendas, the defunding of important social assistance programs, an insistence on fossil fuel development, radical austerity measures, and a rapprochement with oligarchs who choose to publicly support the government, have invited criticism that ‘AMLO can hardly be defined as a progressivist or leftist given, among other aspects, his disposition to follow the economic orthodoxy and his attitude toward social rights’ (Solorio et al., 2021, p. 251) Despite a political discourse that pledges to be ‘the answer to neoliberalism’s failures’, inconsistencies between discourse and political action in areas such as civil, social, environmental, and indigenous rights, have made scholars wonder whether Morena may yet turn out to be ‘the resurrection of the old PRI’ (Rodriguez Nuñez, 2019, pp. 37, 39). It must be pointed out, however, that the Morena movement has managed to maintain overwhelmingly positive results in public polls, that unlike the populism of the PRI era, were developed through clean elections. Finally, the post-transition era has been characterized by the gradual disappearance of ‘hard’ state censorship and media interventionism, which was a critical component of the PRI’s grip on power.

The political backdrop is thus, in important ways, also different from the populist episodes in the Americas previously mentioned, i.e., the infamous soft-coup impeachment process of ex-President Dilma Rousseff that was the entry point for Bolsonarism in Brazil, or the toxification of US politics that culminated in Trump’s election. The election of the Morena government was, without a doubt, a clear mandate from the Mexican electorate that change in Mexican politics at the highest level was desperately required and was certainly not delivered by either the PAN or post-transition PRI presidencies. In this sense, the Mexican government sits in a middle ground between what Levitsky and Loxton (2013) define as ‘full populist’ – led by outsiders to an existing system, such as Morales, Chávez, Perón or Fujimori, that seek to do away with institutional resistance by attacking the system holistically – and ‘maverick populists’ who, forming part of the political system, then turn to personalistic rule without seeking to necessarily overturn institutional life.

## Conceptualizing populism

Work in anthropology and political science has recently pointed out that populism’s expression encompasses a diverse family of political practices and discourses that escape any essentialist definition (Bruhn, 2012; Mudde & Rovira Kaltwasser, 2017, 2018; Rodríguez Sáez, 2021; Rovira Kaltwasser et al., 2017). However, scholars consistently stress the significance across forms of populism of projects for *total organic change*, particularly of institutional life. Meanwhile, as Mazzarella (2019) observes, populism transcends ‘anti-democracy’ and, perhaps surprisingly, ‘charismatic leadership’ as a defining characteristic – Basurto (1969), for example, found that by the 1960s Mexico, the ‘charismatic leader’ or *caudillo* was an exception from the early post-Revolutionary period, growing increasingly rare as the PRI evolved. Rather, Mazzarella (2019, p. 47) posits, populism is a *style* of politics held together in discourse by ‘a Manichean division of the population into a valorized majority us – the people – and a demonized minority them …’ along with ‘a suspicion of high-flown expert discourse and cosmopolitan rootlessness; and a powerful impulse toward bypassing mediating and moderating institutions and procedures in pursuit of an immediate, redemptive and affect-intensive presencing of popular sovereignty’.

**Figure 1.**
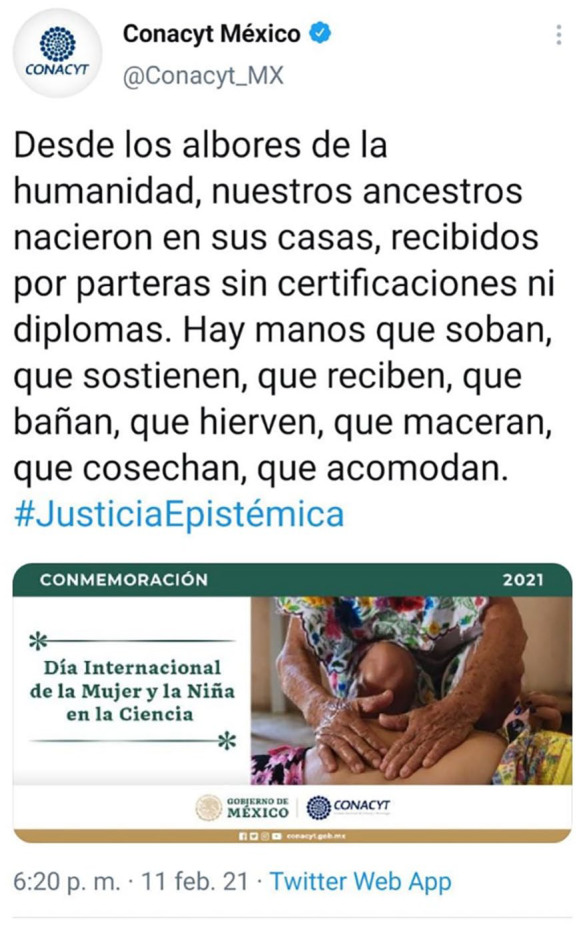
Tweet from the official Conacyt account on the International Day of Women and Girls in Science (11 February 2021), showing the hands of a ‘partera’, or traditional midwife. The tweet reads, ‘Since the dawn of time, our ancestors were born in their homes, aided by *parteras* without certificates or diplomas. There are hands that caress, hold, receive, bath, boil, macerate, reap and accommodate. #EpistemicJustice.’ The tweet was widely circulated and strongly criticized in the media and Twitter. A significant number of replies were by women natural scientists, many arguing that on a date that was meant to celebrate female scientists, ‘evidence-based science’ was being displaced by non-scientific practices, superstitions, and an outdated imposition of traditional women’s roles. It would not be until October of that year that Conacyt would answer these critiques with tangible actions, including discussion forums on the difficulties of traditional and indigenous midwifery (Conacyt, 2021b). See also Argüello-Avendaño and Mateo-González (2014).

In Latin America, ‘to invoke *el pueblo* is to invoke the popular sectors, which include a mix of subaltern groups, most notably the urban masses and rural campesinos’ (Samet, 2019). Morena’s relationship to indigenous movements offers an illustrative example of the complexities and contradictions involved in the government’s self-branding as champion of both *el pueblo* and also the poorest classes and indigenous communities. Moreover, the national STI law’s discursive vindication of indigenous knowledges and the use of concepts linked to indigenous scholarship, such as *diálogo de saberes*, is at the forefront of reforms. One of the proffered reasons for defending the centralization of power in STI decision-making is that ‘traditional’ scientific communities commit epistemological injustices against these communities through their exclusion from scientific policy and practice, and that the ‘good government’ is the sole agent of change capable of overcome these structural injustices (see Figure 1).

An analysis of the political situation at large, however, casts doubts on how legitimate the project to achieve ‘epistemological equity’ really is, particularly considering Conacyt authorities are neither representative nor inclusive of indigenous voices. Also, unlike other populist movements in Latin America in the 20th century, most visibly Bolivia’s (Andreucci, 2019), Morena’s agenda-setting does not stem from indigenous movements themselves, but from a centralist government’s vision of how the indigenous must fit into *its* nationalist agenda. There is no evidence, from available Conacyt documents, of there being significant indigenous community input during the consultation process for the new STI law. Wrapped up in multi-culturalist talk, this *indigenismo* shapes government discourse, but also draws tension between a nationalist agenda driven by ‘the people’, progress, and the interests of the *pueblos originarios* (Ramos, 2019) Consider, for example, AMLO’s overhaul of the stiff corporatist image of Presidential inaugurations in Mexico, when he was handed the *bastón de mando* (ruling staff) that forms an important protocolary element of indigenous power-transfer ceremonies. In contrast, months before he entered power, the Ejército Zapatista de Liberación Nacional (EZLN) – by no means representative of all indigenous communities in Mexico, but arguably the most vocal indigenous political actor – had already published several communiqués stating their strong opposition to the Morena project (EZLN, 2018a), while EZLN figurehead Subcomandante Galeano (formerly Marcos) had strong words for the incoming president in an abrasive public letter (EZLN, 2018b):

No, [we] Zapatistas DO NOT adhere to the campaign ‘for the good of everyone’, for putting ‘the famished first’. You can change the foreman, the slave driver and the overseer, but the slave owner is still the same.^6^

Zapatistas and other indigenous collectives thus maintain a stalwart opposition against some of the administration’s key ‘mega-projects’, mainly, the *Tren Maya* and the *United States–Mexico–Canada Agreement* (EZLN, 2019; PODER, 2020). Environmental organizations and a UN Human Rights Commission report also point to deficient consultation exercises carried out in indigenous communities as unrepresentative justifications for the mega-projects (UN, 2021).^7^ Indigenous justice rhetoric, but without direct indigenous people’s involvement, is thus used as a discursive tool to impose or justify the government’s agenda on those not aligning to ‘the nation’s’ interests (Solorio et al., 2021)., which in the case of the Tren Maya, was criticized as translating policy into a tool for further colonial violence in the name of social progress (Hernández Castillo & Rueda, 2021).

## Mexican ‘post-truth’ politics

Political identity in Mexico is polarized and follows lines of fragmentation across highly stratified social classes, and only grew stronger as the Covid-19 pandemic progressed (Lucca & Aragon Falomir, 2021). Indeed, the image of AMLO jokingly holding folk-religious amulets before the press to ‘ward off’ Covid-19, his position often bordering on Covid-denialism during the first months of the pandemic, his refusal to follow basic face-covering and social distancing guidelines during public events (Peci et al., 2022; Taylor, 2020), were political statements that nonetheless underwrote the federal government’s controversial strategy of non-testing and minimal lockdown measures which contravened ‘accepted’ consensus (Beck et al., 2020; Capdevielle, 2021), and despite notable efforts to also adopt an open, ‘science-based’ – though partisan – mass media communication strategy (Metcalfe et al., 2020).

As noted in comprehensive policy analyses, excess death figures point to Mexico having one of the highest Covid excess-death and medical population fatality rates globally (Sepúlveda, 2021, p. 117). Though governments across the globe faced extreme uncertainty and have been, to different degrees, criticized for following or not following scientific advice – which was markedly unstable throughout the pandemic (Parviainen et al., 2021) – the Mexican government opened itself to severe criticism from scientific communities when it ‘[e]schewed deliberation and collective decision-making in the appropriate collegiate institutions’. This sits in stark contrasts with, for example, the visible influence of specialist panels for Covid-related decision making recently scrutinized by Evans (2022) in the UK. In the Mexican case, ‘key public health decisions were not systematically submitted to independent supervision, outside expert consultation, and constant revision, so that ‘technical decision-making was hampered by discretionary political interference’, (Sepúlveda, 2021, p. 110).

The point here is not to judge the adequacy of the government’s strategy, but to point to the effects on scientific community sentiment in having sidelined government-independent collegiate scientific advice. Further rifts between government and scientific perceptions were motivated by how ‘[t]he federal government continued to antagonize political adversaries, critics, and broad segments of the scientific and health communities through the emergency… while discretionary command and control [was] replicated in the management of the pandemic’ (Sepúlveda, 2021, pp. 111–112). ‘Civil society and academia played a key role in fact-checking and the analysis of available data, contributing to a better characterization of the real burden of disease’. Regardless of whether the high excess deaths can be traced to the neglect of expert advice, the government’s often cavalier attitude towards scientific opinion coming from outside party circles is constantly manifested as an important factor in the negative sentiment towards the government expressed by the scientific community.

For Collins et al. (2020), populism’s most pernicious effect is its denial of scientific institutions’ role in establishing checks and balances in the realm of knowledge production, so that scientifically informed decision-making through it might be possible – science is thus a soft power in the sense that it must be heard and accounted for, but not necessarily followed when there are sufficient and – crucially – explicit political reasons for disregarding scientific consensus. While relevant to the Mexican case in topics such as the government’s sidelining of independent scientific advice during Covid pandemic, such analyses fall short in conceptualizing scientific populism in Global South and particularly Latin American settings: Mexican science exists in such a precarious state of institutional dependance that it can hardly be an effective check on political power. In previous work I have illustrated how Mexican scientific institutions, conscious of this lack of power and dependency, rarely choose to confront and antagonize the state directly, even when there is clear ethical and epistemic obligation to do so (Reyes-Galindo, 2017).

The government’s indifference to collective scientific input was in parallel to its disdain for organized civil society working outside of Morena’s direct control. AMLO’s daily conferences – the *mañanera* soliloquies, as they have been named by the press – have grown to include a weekly segment entitled ‘Who is who in this week’s lies’, dedicated to singling out journalists, but also activists, intellectuals, scientists and other public actors, considered to have spoken ‘in bad faith’ about the federal government. Independent journalist Julio ‘Astillero’ Hernández, who is openly supportive of the President, has nonetheless warned, after being singled out in a 2021 *mañanera*, that the government’s aggressive stance towards dissenting, but responsible and non-corporatist free press is an outstanding source of concern (Astillero, 2021). While Astillero has vehemently denied media accusations of outright censorship, particularly in comparison with the PRI era, press freedom organization *Article 19* – itself already denounced by AMLO as an international ‘conservative’ organization – and other ONGs have pointed out that Mexico remains one of the deadliest places in the world to be a journalist (Agren, 2021a). González-Quiñones and Machin-Mastromatteo’s (2019, p. 668) meta-study of journalistic violence in Mexico concludes that, contradicting the government’s claims, aggression against journalist from organized crime involves ‘around 14% of the cases, while the State is implicated in around 43%’, a view seconded by Arnoldo González and Rodelo (2020).

In this context, the phrase ‘*yo tengo otros datos’* (‘I have other data/facts’) has become one of the President’s catchphrases when confronted by external criticism, for example, in confronting the situation in the fight against corruption that is at the discursive vanguard of government actions, including many of the scientific defunding decisions. Corruption is persistently framed by the government as a moral sickness of the evil upper classes, including the intelligentsia that benefitted from the old oligarchies, and is addressed through the centralization of power into the hands of the ‘good government’. As a systemic problem of great historical complexity for the country (Morris, 1999), by framing corruption as ‘bad acts by bad people’, and then selectively enforcing corruption policies against political rivals, little change has come about. Despite the government often insisting that ‘corruption has ended’, it remains entrenched across all Mexican social life and as Transparency International (2019a, p. 4, 2019b) notes, corruption remains ‘multifaceted and widespread, constituting the most concerning societal problem for Mexicans’, with measured corruption rates ‘may actually be much higher due to low levels of crime reporting’.

## Populist science policy: Reforming the moral values of science

The federal government’s pledge of social transformation through the establishment of new moral principles mixes both nationalist sentiment and historical revisionism, and despite praising secularist heroes of the national pantheon, has religious overtones that additionally reference an unrealistically idyllic past (Capdevielle, 2021). *Morena* (an acronym for *Movimiento de Regeneración Nacional*) alludes both to the Mexican ‘mestizo’ ideology of post-Revolution Mexico (‘morena’ is a generic name for a dark-skinned woman), as much as to Mexico’s patroness, the Virgin of Guadalupe, or *La Virgen Morena*. This framing of transformation is critical for understanding the modifications to the Mexican scientific system.

It was as part of this political and moral overhaul that AMLO selected María Elena Álvarez-Buylla – an accoladed geneticist from the National Autonomous University of Mexico (UNAM) – as Conacyt director, a post equivalent to Minister of Science. Though part of the scientific community saw her research credentials as a positive factor, there were also worries; Wade (2018) documents that by the time she had publicly accepted the directorship, public petitions were being signed opposing her designation due to her staunch ‘opposition to genetically modified (GM) maize’ as well as her ‘plans to open dialogue between Mexico’s scientific community and Indigenous knowledge producers’; some feared this would negatively affect basic research because of ‘Álvarez-Buylla’s professed commitment to science that helps solve societal problems’. This activism, centered on the protection of indigenous maize heritage against the powerful international agricultural lobby (Bonneuil et al., 2014), as well as a strong preference for ‘socially pertinent’ science, was a clear fit for the incoming government’s nationalistic agenda.

Once entering power, the new Conacyt directorship quickly announced important institutional changes, announcing on Twitter a meeting of the General Council for Scientific Research, Technological Development and Innovation (*Consejo General de Investigación Científica, Desarrollo Tecnológico e Innovación*) headed by the President and resulting in an Executive order for Conacyt to draw up a blueprint (*anteproyecto*) for a General STI Law (Crónica, 2019). The meeting, quite controversially, did not include the legally mandated participation of a senior scientific advisory group to the Presidency, the *Foro Consultivo Científico y Tecnológico* (*FCCyT)*, which vocally protested on Twitter. Since 2006, the FCCyT had operated as a civil association – as mandated by the still current National Science Law and Conacyt’s organic bylaws – acting as an autonomous consultancy group for top-level STI policymaking, for input on policy from the scientific community. The General Council meeting that had been held, on the other hand, had included exclusively political players, and only a handful of academic actors closely linked to the federal government’s intellectual circle. The hashtag with which the FCCyT ended its tweet included *#GobernanzaIncluyente* and *#ComunidadCTI*, alluding to the lack of representation of scientific opinion in the policy process (FCCyT, 2020). Indeed, a federal judge had, by July 2020, emitted an *amparo* (habeas corpus) order that mandated Conacyt to include the FCCyT in top policy meetings – a decision that Conacyt refused to follow, apparently without tangible consequences. What had begun as a discussion on the legality of the FCCyT’s exclusion quickly soured into the overtly political. In response to the court mandate, Conacyt instead financially crippled the FCCyT and created a ‘parallel’ Foro, discrediting the former’s members in public by claiming that the Foro had usurped Conacyt’s consultancy roles,^8^ and finally associating members of the FCCyT with acts of corruption.^9^

### Conflict and changing values

While the federal government’s alienation of scientific collegiate bodies from pandemic-related discussions and science policy circles had already put many Mexican scientists on alert (Pérez Ortega & Wessel, 2020), when drafts of the STI law project were leaked online, there were additional causes for concern. The reframing of the ‘principles’ that would underpin the new law were perceived by many as especially controversial. One paragraph in particularly was read to undermine basic or non-applied research, in favor of what many natural scientists saw as ‘pseudosciences’:

The human right to science will be exercised adhering to the principles of epistemological rigour, equality and non-discrimination, inclusion, plurality and epistemic equity, diálogo de saberes, the horizontal production of knowledge, collaborative work, solidarity, social good, and precaution. (Conacyt, 2020b, p. 11)

Currently, most Conacyt institutional communications are begun or include in the body this or a slightly modified version of this statement.

The rejection of this by a significant proportion of Mexican natural scientists was clearly evidenced in both forums dedicated to the law’s discussion. The natural sciences, on the one hand, considered plurality, epistemic equity and equality as part of an ideological deviation from ‘Western’, value-free science. For this side, the new values not only undermined ‘science’, but would make Conacyt ‘anti-scientific’. The humanities, on the other hand, defended changes to the traditional values precisely to counterbalance the effects of ‘Western’ monolithic views that overwhelmed those of ‘traditional’ knowledges.

Yet, as independent analyses of the full law project have shown, the actual implementation of regulations makes little use of the proposed ‘new values’ and, instead, in the body of the law goes on to establish highly centralized and vertical governance structures, with the President of the Republic as the controversial final arbitrator in matters of national science policy (Arámburo de la Hoz, 2021; Gutiérrez Jaber, 2021). During one of the discussion forums regarding the Law project, Dr. Rosalba Casas, a leading Mexican STS scholar, stated about the Law project:

The conceptualization [of governance] is centralist, hierarchical, and based on government decisions …. The project ignores the various diagnostics that have pointed to the construction and coordination of governance as central to a system of science, technology and innovation. … What can be deduced from this law project is that it will be the State’s agendas and politics that will define how ‘indispensable technology for the country’s development’ is defined. … Given that participatory mechanisms are not part of this proposal … the panorama is that scientific, social scientific and humanistic knowledge created within universities will linger on, in a grander scheme of discretionary interests (ProCienciaMX, 2021b).

The *ProCiencia* network, an academic grassroots civil organization that has been the only sizeable counterbalance to Conacyt and was influential in setting up the first forum, stated in a press release regarding the Law project:

The draft legislation emphasizes that the State’s Development Agenda should be thoroughly permeated by ‘epistemic equity’, that is, by equity in the many and diverse forms of producing and organizing knowledge that are generated from within ethnically diverse sectors of our nation’s society. … Without a doubt, ‘diálogo de saberes’ plays an important role in many topics covered by the sciences and the humanities. Nevertheless, by supposing such a dialogue to be transversal, the draft legislation disregards the diverse disciplinary and thematic courses that scientific activity moves through, both around the world and in our country. These not only demand the development of basic science with freedom of research without blinders, but also that it be based on an epistemological rigor … that often plays no part in dialogues with knowledges that are culturally or ethnically determined. (ProCienciaMX, 2021a).

Given the antagonisms between the federal government and natural scientists that had been set in motion with the Foro’s dismissal and was strengthened with the pandemic responses and other policy decisions, the centralization of power and the imposition of a government agenda appeared to be legitimate concerns for the Mexican scientific community. Thus, the increasing calls for the respect of scientific autonomy and ‘freedom of research’ cannot be offhandedly dismissed as simply a naïve appeal to objective, universal, value free science (Lacey, 2005), but *also* as a response to the imposition of outside governance norms within an overtly hostile political environment. Government sympathizers, such as traditionally left-leaning and (now) pro-government newspaper *La Jornada*, nevertheless considered all anti-Conacyt reactions as racist, classist and ‘epistemically sexist’ commentary (Hernández García, 2021), arguing that in Mexico researchers can ‘be part of the National Researcher System, teach in a university, have [sic] a postdoctorate, and at the same time have a myopic and reduced vision of the country’s cultural diversity’, because ‘the hubris of great academics allows it’.

## The ‘end’ of STI corruption in Mexico

The polarizing nature of the new law’s framing seems odd once the process that led to its development is scrutinized, as both in paper and action it appeared committed to capturing a wide spectrum of STI stakeholder opinions. The drafting was preceded by a wide consultation exercise organized by Conacyt, including numerous round tables, open parliaments, expert presentations and scientific community-engagement events. That these opinions were largely ignored, in fact, is one of the fundamental aspects of natural scientists’ distress, including many who participated in those consultations.^10^

Developments in funding policy near the end of 2020 also influenced the scientific community’s concerns about the direction the national STI system was taking. The most politically charged was the breakneck dissolution of a set of important funding channels for science: *fideicomisos* (public trusteeships), widely used in large-project financing within the public sector. These were the heart of Conacyt’s sectoral funding programs, provided year-to-year funding stability, and allowed Mexican public institutions to capture and autonomously manage scientific funding from abroad. Required to do public reporting, they were also seen as assuring some measure of public accountability. Yet the fideicomisos’ dissolution, despite notable protests from the scientific, academic, and artistic communities they directly affected, gained fast-track approval from both chambers of the government-controlled legislature: 109 fideicomisos were ended, comprising US $3 billion in funding, including 65 Conacyt programs (Barrón, 2020). The government spun the decision as a measure in the fight against corruption in the STI system. Conacyt audits, it publicly declared, had revealed massive transfers of public funds to private enterprises that had provided little value for money. While the government announced that evidence for corruption would be released to the public immediately, and AMLO himself pledged that within ten days a brief on each fideicomiso would be provided (Aristegui Noticias, 2020), that promise was (and to date remains) unfulfilled, even as the President continued to denounce critics of this measure as ‘conservatives’ interested in preserving the old oligarchic networks:

They [critics in Congress against fideicomiso measures] try to pass themselves off as the people’s paladins. But no, what they’re really defending is the corrupt regime that existed, and that we want to eradicate completely. Of course, we will show how the fideicomisos operated. Of course, we will do it, answer back to all of them (they who spend every night trying to hinder, to impede, reforms that will end corruption) so that those funds will benefit the people [El Pueblo].

Then turning his guns to increasing criticism from the scientific community, AMLO further demonized them as examples of reactionary conservative sectors that had long-abused public funds and were afraid of losing their privileges:

They worry a lot about science and technology, because they will lose all support for these matters … they try to give the impression of being the people’s champions, but no, what they are doing is defending the corrupt regime that existed, and that we are trying to do away with.

Despite the enormous impact on Conacyt funding, days later Álvarez-Buylla made an appearance during the *mañanera* to support the cuts: Audits ‘revealed’ that 44% of Conacyt funding through fideicomisos and tax-break programs, totaling around US $2 billion, had been assigned to ‘unaccountable’ private companies, such as Volkswagen, Intel, Bayer, Monsanto and IBM. Such funds were hard to justify, according to Álvarez-Buylla, in ‘real, scientific terms’. However, given that only 24% of fideicomiso moneys were assigned to the private sector, the dissolution of fideicomisos *also* eliminated the country’s most important instrument for publicly funded science. Conacyt’s discursive focus nevertheless remained on the ‘opaque’ transfer to the private sector, including private universities, as well as increasingly harsh accusations of corruption against former Conacyt management. When later questioned about allegations that she herself had received over US $800,000 of funding through fideicomisos, Álvarez-Buylla replied that these funds had been obtained through open call grants, not through ‘neoliberal’ funding:

Neoliberalism installed a funding system for science and other areas and, amongst the instruments that enabled this failed distribution, were fideicomisos, which deposited vast amounts of the people’s resources, instead of assigning them directly to beneficiaries. … Today we are living through a transformation … which privileges the people of Mexico and environmental care in favor of all, and firstly the poorest.

FOI requests showed, however, that Álvarez-Buylla had received funds through two of the instruments she had denounced in her *mañanera* slides, alongside fideicomisos proper. Other prominent academics who were part of AMLO’s inner circle would also be pointed out, through FOI requests and journalist leaks, as gaining advantage from funding favoritism (Sheridan, 2020a, 2020b). Today, many academics consider that the old oligarchic networks, while they exist and continue to dictate Mexican academic life, only appear to be changing hands, now weighted towards those in power and their supporters.

The federal government has been described in the scientific press as having crippled the STI system ‘to a breaking point’ through the enactment of ever more debilitating budget cuts in the name of ‘Republican austerity’, further compromising an already struggling system (Guglielmi, 2019, p. 294; Vera, 2021). Defunding measures, some of them clearly aligned with Álvarez-Bullya’s known aversion to specific research areas, and her political preference for others (mainly, those named in draft versions of the State Agenda), included the removal of biotechnology as a key funding category, which was seen as ‘as an attempt by Álvarez-Buylla Roces, a plant scientist and vocal critic of genetically modified organisms, to control biotechnology research and deprive it of resources’ (Gutiérrez Jaber, 2021). Other defunding or austerity measures included: cutting off funding to learned societies that ‘could lead to a withdrawal from the global science scene that scientists warn will isolate Mexico scientifically and deprive it of opportunities’ (Vidal Valero, 2020); precarization and ‘re-branding’ of the flagship *Cátedras Conacyt* early career researcher placement (ERC) program, including dozens of ERC dismissals that have led to accusations of gender discrimination and more than a hundred lawsuits for wrongful termination, with female researchers denouncing ‘a lack of humanity that is completely impregnated in Conacyt’ (Pérez Ortega & Gutiérrez Jaber, 2021); curtailing major postgraduate study-abroad programs for the natural and social sciences unaligned with the State Agenda; police closure of the private university Universidad de las Américas Puebla^11^; and direct interference in the governance of public research centers.

## Cronyism in the National Research System

The 2021 cancellation of financial stimuli for private university researchers belonging to the National Researcher System (SNI) became another source of bitterness between academics and Conacyt (Dillon, 2021). Initially an honorific recognition for top tier scientists in both the public and private sectors, the later addition of a monthly stipend for public university researchers turned it into an indirect fix for deteriorated salaries in public universities, but had been recently extended to privately employed researchers (Didou Aupetit & Gérard, 2010). It is generally agreed that being a member of the SNI is a highly significant research-career achievement, with the Tier 2 and 3 categories demanding high quality publications in journals of international standing, wide teaching experience – a SNI 3 being the hallmark of an especially distinguished research career.

In mid-2020, the Mexican academic community would be further rocked by press reports that Mexico’s Attorney General, Alejandro Gertz Manero, had been awarded the Tier 3 SNI distinction. Academic uproar was followed by journalistic scrutiny of the career politician’s academic production, which was perceived by most academics as definitively lacking enough quality for SNI membership – let alone a Tier 3 award. Adding fuel to the fire, leaked documents showed that the SNI distinction had been awarded by a fit-for-purpose ‘special commission’. After the leaks, the head of the special commission countered that Gertz Manero’s scientific production of ‘five books as sole author’ was of ‘national and international transcendence’, with the motley collection of secondhand biographies infamously described by Álvarez-Buylla as ‘notable works’. FOI journalism also revealed that Gertz Manero had attempted and failed to be accepted into the SNI since 2010, having been rejected on four separate occasions by large peer-review committees, with two of these re-evaluations only forced upon past SNI administrations by court challenges promoted by Gertz Manero. A fifth re-assessment had then been ordered by the National Commission for the Prevention of Discrimination (Conapred), after which the ‘ad hoc’ commission (unprecedented in SNI history), finally awarded the SNI 3 distinction – also in disregard of SNI rules, which state new members must progress through all the ranks to reach the higher tiers.

The coda to the SNI 3 *telenovela*, however, did not fail to impress: only a few weeks later, public intellectual and literary critic Sheridan (2021) publicly offered unambiguous evidence that several of the books of ‘international transcendence’ had in fact been plagiarized by Gertz Manero. The public release of the minutes from the ad hoc commission that has been demanded by many academics and SNI members has furthermore been met with absolute institutional silence. Finally, in March 2022, the SNI’s Honor Commission unanimously decided to dismiss an official complaint, signed by 250 SNI members, documenting the accusations of plagiarism and demanding a full ethics and scientific integrity query into Gertz Manero’s award. The grounds for the dismissal – that none of the authors of the books was signatory to the complaint – was taken as a further sign of the Conacyt’s failing integrity, all the plagiarized authors being, after all, long dead.

## Criminalizing scientists

The FCCyT’s demand to be included in Conacyt’s policy processes during the current directorship’s first weeks in power has cut a heavy cost on its members. On 20 September 2021, journalist Riva Palacio (2021a, 2021b) revealed that the newly appointed SNI 3 Attorney General had requested an arrest warrant of ‘31 Mexican scientists’, which included former members of the FCCyT and Conacyt ex-management and administrative staff. The accusations were of the utmost gravity: misappropriation of public funds and organized crime. The latter could potentially put the ‘31 scientists’ inside a high security federal penitentiary, in what was read as a personal vendetta on non-aligned scientific community figureheads because of growing opposition to the Attorney General’s SNI 3 appointment, as well as Álvarez-Buylla’s determination to see the FCCyT undone at any cost. Gertz Manero’s Federal Prosecutor’s Office (FGR) reiterated it would continue pressing the case, while Conacyt bombarded SNI members with emails and corporate videos trying to distance itself from the legal procedures it had, in fact, set in motion:

Holding to the principles of Republican Austerity, ethics, transparency, and accountability, Conacyt offered [the FCCyT] modest resources, but enough for substantial activities, which were rejected … In 2019, Conacyt handed in evidence of misappropriation of funds, and that was the end of its involvement. Without denouncing any persons. Take note, without pointing out names, or suggesting crimes. … The legal process is not directed against scientists in their role as scientists. … Conacyt does not persecute scientists, on the contrary, it supports them. (Conacyt, 2021a, author’s transcription)

In contrast, only days later, the President jeered the ‘31 scientists’, accusing them of absurd spending on luxury restaurants, using chauffeurs, and having bought ‘a luxury house’ in southern Mexico City (Arista, 2021):

They are a group favored by the previous regime, and since now they can’t keep their privileges, they feel persecuted. … I ask the people, is the fight against corruption going to be selective, or is it going to be even? Are we going to exclude the powerful, the elites from academia, from science, intellectuals, economic elites, or are we going to combat corruption all out?

Further on, he commented how the old regime’s scientists frivolously spent money, compared to the ‘new’ Conacyt’s devotion to austere applied research, and then painting run-of-the-mill scientific spending as excesses in luxury:

They complain that we don’t invest in science and that we don’t care about technological innovation. What did they do? Nothing: colloquiums, congresses, trips abroad, expenses. Well, not now. Now it’s applied research. Conacyt has done extraordinary things: produced ventilators for Covid patients, the Patria vaccine, which is seeing good development. (Financiero, 2021a)

In fact, AMLO has a remarkable history of both misunderstanding and misrepresenting the scientific community. In one mañanera, for example, he turned to historical revisionism to ask ‘who it was that had supported the [dictatorship] of Porfirio Diaz?’ in the late 19th century. His answer:

Well, ‘The Scientists’ [‘*Los Científicos’*]! ‘The Scientists’! That is how they were known… not everyone who is involved in science, not everyone who works in culture, in research, in academia, is a conscientious person.

This was a deeply symbolic equivocation. ‘The Scientists’ (‘*Los Científicos’*) were a late 19th century group of European-educated, positivist technocrats who had, indeed, been deeply influential in supporting the Diaz dictatorship, while some had also been instrumental in the establishment of Mexican public education (Priego, 2007). Importantly, by linking ‘the scientists’ to the Diaz dictatorship, a rhetorical link could be established between today’s ‘scientists’ and the ‘conservatives’ of the Porfiriato.

The legal procedures that continue against the ‘31 scientists’ have created some waves in the international press, but remain mostly unknown outside of Mexico (Agren, 2021b). Still, major academic institutions in Mexico and abroad, learned societies, universities and professional associations, including the National Human Rights Commission, have made public their concern about the persecution of academics by the Attorney General’s office, particularly since ‘such charges are typically reserved for narcotics traffickers, and are so serious that even just a formal accusation can result in incarceration in a maximum-security prison without the chance of bail until a trial is held’ (Reardon, 2021). Morena Senator Jose Guadiana, in addition to defending the persecution of the ‘31 scientists’, proposed that similar investigations into academic spending should be extended to all publicly funded universities, though the call had almost no resonance within the party and was dropped (García, 2021). Meanwhile, Conacyt (2020b) continues to hold that criticism is ‘an orchestrated wave of disinformation in the media and social networks’, even though high-ranking Morena have also cast doubts on the legitimacy of the accusations (Sánchez, 2021). AMLO also described UNAM – the standing symbol of the free public university in Mexico – as ‘a defender of neoliberal projects’ that requires being ‘shaken up’, closely following the election of one of the ‘31 scientists’ into the UNAM General Council, its highest-ranking collegiate body (Financiero, 2021b).

## Discussion: Understanding the Mexican STI reforms

How legitimate is the concern of Mexican scientists and academics about the value changes proposed by Conacyt? How is STS relevant to these debates, given both its historical preoccupation with promoting alternate forms of scientific governance to that of autonomous science, and its recent concerns about the influence of populist regimes in the ‘post-truth age’?

The episodes described here are consistent with Mede and Schäfer’s (2020, p. 482) observation that in confrontation between populism and science, the former typically uses two fronts to undermine science’s legitimacy. First, populists focus on *decision-making sovereignty*, which is ‘the authority over decisions about what is being, or should be, researched when, how, and by whom’ and additionally deny science truth-speaking sovereignty, or ‘the authority over defining what constitutes “true” knowledge’. As Mede and Schäfer (2020, p. 483) explain, populists typically argue that science is illegitimate because it is guided by the interests of an ‘insiders club’ that is corrupted by desire for personal gains and selfish political interests, so that ‘legitimate possessors of science-related decision-making sovereignty should be the ordinary people – because their ideas about the who, how, and why of scientific knowledge production are allegedly not biased by elite interests’. This strategy has been used by the Mexican government to severely defund any academic fields not aligning with the State Agenda, which is in turn manufactured squarely within party circles. On *epistemological sovereignty*, populist interventions often maintain that scientific knowledge is on par with ‘other’ knowledges (such as those of the *pueblo bueno/sabio* – the good/wise people – another key AMLO catchphrase), so that science is illegitimate ‘because scientific approaches to knowledge production do not prioritize the everyday experiences and opinions of ordinary people’ (Mede & Schäfer, 2020, p. 483). As shown, this argument has been used by Mexican authorities to reorient the values of the national scientific system by bringing in political talk about ‘traditional’ knowledges and epistemologies into the spotlight of STI governance, but without any participation of either the stigmatized groups they claim to represent, or of the affected scientific communities. Conversely, the change of values talk was used as the justification for appropriating massive funds from the scientific system, which were then moved to support the State’s infrastructure projects in a moment of economic uncertainty – including a new international airport, an oil refinery, and the Tren Maya, as recently admitted by Alvarez-Buylla herself before a special Senate commission (Cruz, 2022).

In their reflection on how STS could benefit from thinking about science ‘in the South’, Dumoulin Kervran et al. (2018, p. 294) note that ‘the vocabulary, concepts and theories of STS are circulating throughout the world, mobilized by social movements and intergovernmental organizations that recycle studies on the co-production [of] science/society, aiming to make “epistemic plurality” part of their thinking’. Thus, concepts widely appreciated in STS are explicit in the discussions of values and the ongoing conflicts between Conacyt administration and the Mexican scientific community: epistemic injustice (Fricker, 2007; Santos, 2015) versus scientific objectivity, social responsibility in research (Owen et al., 2012) versus scientific autonomy, diversity and inclusion in knowledge creation (Harding, 2015) versus the universality of knowledge, the democratization of science (Callon et al., 2011) versus expertise-based science, wider representativity and pluralism in STI systems (Kellert et al., 2006) versus scientific self-governance, and indigenous and ontological politics (de Castro, 2015) versus the centrality of Western perspectives in science, would seem to be addressed by the proposed value changes. The inclusion of *diálogo de saberes* in a national STI law, for example, can be read as a progressive policy akin to the recent codification of Rights of Nature/*Buen Vivir*/*Sumac Kawsay* into the Ecuadorian constitution or the inclusion of Maori perspectives into environmental policy in New Zealand (Kramm, 2020), but has instead been criticized as an attempt to undermine ‘real’ science in favor of a nationalistic agenda.

Yet as Dumoulin Kervran et al. (2018, p. 288) also point out, a limitation of STS engagements with ‘the South’ has been their limited capacity for ‘asking exactly how to label and analyze the forms of government and the ways in which power is imposed and perpetuated – or challenged’ outside the (Northern) scientific centers of production. Thus, epistemic values that might function as toolkits for transformative agendas in science policy in a Northern STS setting, may become imposed as norm-violating principles for political interference in a setting like Mexico’s. Here I have illustrated how the transformations proposed for the STI system indicate an *organic shift* allegedly based on values that are, in their present form, an imposition on the scientific community, even if certain academic sectors highlight positive aspects. Meanwhile, Conacyt actions point to a replacement of the status quo with party insiders, rather than actual attempts at facing the governance or structural issues that affect the country’s science. The alleged fight against corruption spearheading many defunding actions stands in stark contrast with the public scandals of cronyism involving the current directorship, which even typically pro-government academic actors have largely failed to support. The form of science governance put on the table is, however, consistent with the political situation at large in Mexico: the centralization of power into the hands of party-friendly groups; demonization of party outsiders; sentimental appeals to an idyllic past in nationalist ahistorical narratives; and appropriation of the social claims of stigmatized populations to spin a political agenda that does not address epistemological injustices, but rather consolidates State power.

While the proposed change in values to Mexican STI policy might appear consistent with democratic-turn STS insights and projects for a more pluralist academia, in practice these may be only acting as sweet coatings for the legitimization of arbitrary institutional violence against scientific communities and individuals outside the ruling circles, and for the appropriation of funds from science and education to more (short term) politically profitable infrastructure projects. Younger and less established researchers are being affected in the short term by unprecedented levels of precarity. The reforms recall Valiverronen and Saikkonen’s (2021, p. 12) analysis of the effects of populism in the Finnish academic context which, as the Mexican case, illustrates ‘how populist tendencies can entail a distancing from researchers’ views and can portray researchers as elites who are alienated from the world’, thus weakening scientific, academic and intellectual voices that can confront populist claims. Here I have tried to contextualize the grievances against Mexican academic communities in what I propose is a form of ‘trickle-down populism’ that has, in effect, resulted in an imposition of a model of *populist science governance*.

## Notes

1. ‘The politician never acts, consciously, against his own interest. What can then be thanked?’

2. One of the first political intervention proposed by Conacyt’s current director was to change the name of the Council to *Conahcyt: Consejo Nacional de Humanidades, Ciencia y Tecnología*, in order to reflect a greater inclusion of humanities and social sciences in the council’s institutional identity.

3. See, for example, Lorenzo Meyer’s denunciation of the Gertz Manero case outlined later in the article, while still demanding a reckoning on the failings and corruption of past administrations. https://aristeguinoticias.com/2709/aristegui-en-vivo/mesa-politica-en-vivo/la-fgr-contra-foro-cientifico-es-un-canon-contra-un-mosquito-por-que-no-va-contra-epn-mesa-de-analisis-video/

4. The recorded sessions are available as the *Jornadas de reflexión sobre el sistema de Ciencia, Tecnología e Innovación que demanda el futuro*, totalling about 25 hours of recorded material. https://prociencia.mx/jornadas-de-reflexion-sobre-el-sistema-de-ciencia-tecnologia-e-innovacion-que-demanda-el-futuro/

5. Session recordings are available as the Foros temáticos nacionales *Hacia la primera Ley General en materia de HCTI en México*, totalling over 30 hours, can be accessed at: https://aristeguinoticias.com/2709/aristegui-en-vivo/mesa-politica-en-vivo/la-fgr-contra-foro-cientifico-es-un-canon-contra-un-mosquito-por-que-no-va-contra-epn-mesa-de-analisis-video/

6. ‘No, nosotras, nosotros, zapatistas, NO nos sumamos a la campaña ‘por el bien de todos, primero los huesos’. Podrán cambiar el capataz, los mayordomos y caporales, pero el finquero sigue siendo el mismo. Ergo…’ Marcos’s words here made reference to the Morena slogan of acting ‘for the poor first’.

7. A good source of documentation can be found in ‘Postura del Centro Mexicano de Derecho Ambiental respecto al Proyecto Tren Maya’, 8 June 2020. https://www.cemda.org.mx/postura-del-centro-mexicano-de-derecho-ambiental-respecto-al-proyecto-tren-maya/

8. See https://www.foroconsultivo.org.mx/FCCyT/boletines-de-prensa/conferencia-de-prensa-fccyt for the FCCyT’s answer to these accusations. The original document could no longer be found in Conacyt’s institutional webpage.

9. See the webpage *La Mafia de la Ciencia* by investigative journalism group PODER which documents the alleged corruption and misappropriation of funds, based on FOI requests, judicial files and interviews with former FCCyT directors, tracing the timeline of both sides’ arguments. https://lamafiadelaciencia.poderlatam.org/

10. See the available documentation at https://consulta.conacyt.mx/?page_id=1233

11. Coincidentally, this was the original host institution for the 2022 *Society for Social Studies of Science’s Annual Meeting*.
